# Determination of Two Differently Manufactured Silicon Dioxide Nanoparticles by Cloud Point Extraction Approach in Intestinal Cells, Intestinal Barriers and Tissues

**DOI:** 10.3390/ijms22137035

**Published:** 2021-06-29

**Authors:** Na-Kyung Yoo, Ye-Rin Jeon, Soo-Jin Choi

**Affiliations:** Division of Applied Food System, Major of Food Science & Technology, Seoul Women’s University, Seoul 01797, Korea; iko0105@swu.ac.kr (N.-K.Y.); yrjeon0715@swu.ac.kr (Y.-R.J.)

**Keywords:** silicon dioxide, manufacturing method, cloud point extraction, fates, intestinal cells, intestinal barriers, tissues

## Abstract

Food additive amorphous silicon dioxide (SiO_2_) particles are manufactured by two different methods—precipitated and fumed procedures—which can induce different physicochemical properties and biological fates. In this study, precipitated and fumed SiO_2_ particles were characterized in terms of constituent particle size, hydrodynamic diameter, zeta potential, surface area, and solubility. Their fates in intestinal cells, intestinal barriers, and tissues after oral administration in rats were determined by optimizing Triton X-114-based cloud point extraction (CPE). The results demonstrate that the constituent particle sizes of precipitated and fumed SiO_2_ particles were similar, but their aggregate states differed from biofluid types, which also affect dissolution properties. Significantly higher cellular uptake, intestinal transport amount, and tissue accumulation of precipitated SiO_2_ than of fumed SiO_2_ was found. The intracellular fates of both types of particles in intestinal cells were primarily particle forms, but slowly decomposed into ions during intestinal transport and after distribution in the liver, and completely dissolved in the bloodstream and kidneys. These findings will provide crucial information for understanding and predicting the potential toxicity of food additive SiO_2_ after oral intake.

## 1. Introduction

Amorphous silicon dioxide (SiO_2_) is widely applied to the food industry as a food additive—for example, as a thickener, anticaking agent, carrier for fragrances and flavors, chillproofing agent in beer, or filter aid [1,2]. As a direct additive, the United States Food and Drug Administration regulates that the level of SiO_2_ cannot exceed 2% by weight of the food, whereas it can be added in the amount necessary to obtain its intended functionality as an indirect additive [3]. It is registered as food additive E551 in the European Union, and its maximum levels in dried, powdered foods and tablet foods are authorized at 10 g/kg and quantum satis level, respectively [4]. An acceptable daily intake for SiO_2_ is not currently specified [5,6]. Until now, food additive SiO_2_ has been used for a long time without any adverse effects on human health at its levels of usage. However, the European Food Safety Authority (EFSA) suggests re-evaluating the toxicity of E551, due to the presence of nanosized SiO_2_. A nanomaterial is defined as a material with more than 50% of its particles having at least one external dimension in the size range of 1–100 nm, based on number size distribution [7]. The EFSA report and other studies demonstrated that nanoparticles (NPs) of less than 100 nm were present in E551, although the amounts of NPs differed between the materials tested and analytical methods [8,9,10]. Recently, it was reported that E551 can be considered to be a food additive intentionally produced to nanosized materials [11]. However, current specifications do not include the particle size distribution nor the percentage of NPs in SiO_2_ [3,8].

Concerns about the potential toxicity of SiO_2_ have been raised because some in vitro studies have suggested that SiO_2_ NPs can cause inflammatory responses of the intestinal wall [12,13] and affect small intestinal function [14]. Neurotoxicity of SiO_2_ NPs was also reported in neuronal cells and zebrafish [15,16,17]. However, there is no evidence that these toxicological findings are comparable to in vivo animal models and humans. Indeed, the no-observed-adverse-effect level (NOAEL) of SiO_2_ NPs was reported to be more than 2000 mg/kg [18]. Meanwhile, SiO_2_ is known to not be rapidly decomposed into Si ions under acidic or biological conditions [19]; it undergoes in vitro and in vivo gradual degradation, forming silicic acid (ortho-, meta-, di-, and trisilicates) via hydrolysis [20,21]. Therefore, the determination of the fates of food additive SiO_2_ in commercial products and biological systems is of importance, and can provide information about the presence and amount of NPs, aggregates, or dissolved forms, contributing to the understanding of its potential toxicity.

Another factor to be considered is that the physicochemical properties and biological responses of SiO_2_ can be also influenced by the manufacturing method. Indeed, commercially available food additive SiO_2_ is manufactured by two different methods: One is precipitated SiO_2_, produced by the reaction of metal silicate solutions and sulfuric acid, resulting in white precipitates [22]. The other is fumed SiO_2_—also known as pyrogenic SiO_2_—which is produced by the flame pyrolysis of silicon tetrachloride (SiCl_4_), or from quartz sand vaporized in an electric arc at 3000 °C [23,24]. It was reported that the dissolution and physicochemical properties of SiO_2_ NPs differ between the two manufacturing methods [25,26]. Hence, the fates of precipitated and fumed SiO_2_ NPs in the body, which have not been clearly demonstrated yet, might be also different.

In the present study, we developed a cloud point extraction (CPE) approach to separate SiO_2_ NPs captured in Triton X-114 (TX-114)-based micelles from biomatrices. TX-114 is a cost-effective surfactant, and forms micelles at room temperature. CPE methods using TX-114, ethylenediaminetetraacetic acid, or 8-hydroxyquinoline were used to analyze trace NPs under aqueous conditions or minerals in tissues, resulting in their separation in the precipitated, surfactant-rich phase [27,28]. In this case, pretreatments to digest organic matrices were essentially required prior to CPE application. However, pretreatment with acids at high temperatures can decompose or dissolve NPs into small molecules or ions, thereby inhibiting NP detection as an intact particle form. No attempt has been made to detect SiO_2_ NPs in complex biosystems using a CPE approach. Optimization of the CPE method depending on matrix type, such as cell and tissue matrices, was carried out in order to determine the fates of SiO_2_ NPs in intestinal cells, intestinal barriers, and tissues after oral administration in rats. Two differently manufactured food additive SiO_2_ NPs—precipitated and fumed types—were used to investigate the effects of the manufacturing method on the particles’ fates in biological systems.

## 2. Results

### 2.1. Characterization

**Figure 1:**
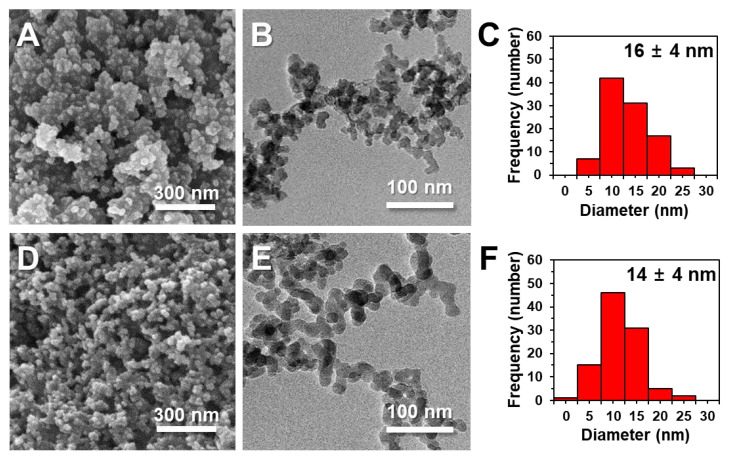
(**A**,**D**) Scanning electron microscopy (SEM), (**B**,**E**) transmission electron microscopy (TEM), and (**C**,**F**) size distribution of (**A**–**C**) precipitated SiO_2_ and (**D**–**F**) fumed SiO_2_. Size distributions were obtained by randomly selecting at least 100 particles from the TEM images.

The constituent particle sizes and size distributions of two differently manufactured SiO_2_ particles were measured via scanning electron microscopy (SEM) and transmission electron microscopy (TEM). Figure 1 demonstrates that the constituent particle sizes of precipitated and fumed SiO_2_ particles were 16 ± 4 and 14 ± 4 nm, respectively, with round shapes. No significant difference in average size between the two particles was found (*p* > 0.05). The dynamic light scattering (DLS) results in distilled water (DW) show that the particle fraction of fumed SiO_2_ was smaller (43% smaller than 100 nm) than that of precipitated SiO_2_ (96% larger than 200 nm), based on number % (Table 1). The Z-average diameters of precipitated SiO_2_ (375 ± 1 nm) were larger than those of fumed SiO_2_ (156 ± 1 nm), indicating that the former was more aggregated than the latter in DW. On the other hand, the specific surface areas of precipitated and fumed SiO_2_ particles, as measured by the Brunauer–Emmett–Teller (BET) method, were 167 ± 1 and 177 ± 1 m^2^/g, respectively (Table 1). The zeta potential values of both SiO_2_ particles as a function of pH are presented in Figure S1A, showing more negative charges for precipitated SiO_2_ than for fumed SiO_2_, at pH levels ranging from 2 to 5. The isoelectric points (IEPs) of precipitated SiO_2_ and fumed SiO_2_ were pH 1.53 and 1.92, respectively. The hydrodynamic diameters of SiO_2_ particles were also measured as a function of pH, showing high aggregation of both particles at low pH (below 3.0) (Figure S1B). Smaller hydrodynamic diameters were also found for fumed SiO_2_ than those for precipitated SiO_2_ at pH 2.0–7.4 (Figure S1B).

### 2.2. Method Validation and Quantitative Analysis in Cells or Tissue Matrices

The accuracy and precision of the analytical procedure for SiO_2_ were explored using Si standard solution, precipitated SiO_2_, fumed SiO_2_, and SiO_2_ spiked with human intestinal Caco-2 cells or liver tissue, after predigestion with nitric acid (HNO_3_)/hydrofluoric acid (HF) in a microwave digestion system, followed by inductively coupled plasma atomic emission spectroscopy (ICP-AES) analysis. Indeed, HF is effective at dissolving SiO_2_ into Si ions [29], while HNO_3_ is widely applied to digest organic matrices [10,29]. Table 2 shows that the recoveries (%) of Si standard solution, pristine food additive SiO_2_, and SiO_2_ in spiked cells or liver tissues ranged from 96.1 to 105.0%, with repeatability (relative standard deviation, RSD%) of 1.54–5.12%. The limit of detection (LOD) and limit of quantification (LOQ) values of SiO_2_ in cells or tissue matrices were highly sensitive, showing 0.36–0.52 SiO_2_ μg/g of matrix and 1.08–1.58 SiO_2_ μg/g of matrix, respectively. The root-mean-square error (RMSE) values were also small, indicating good quality of the quantitative results obtained.

### 2.3. Solubility

**Figure 2:**
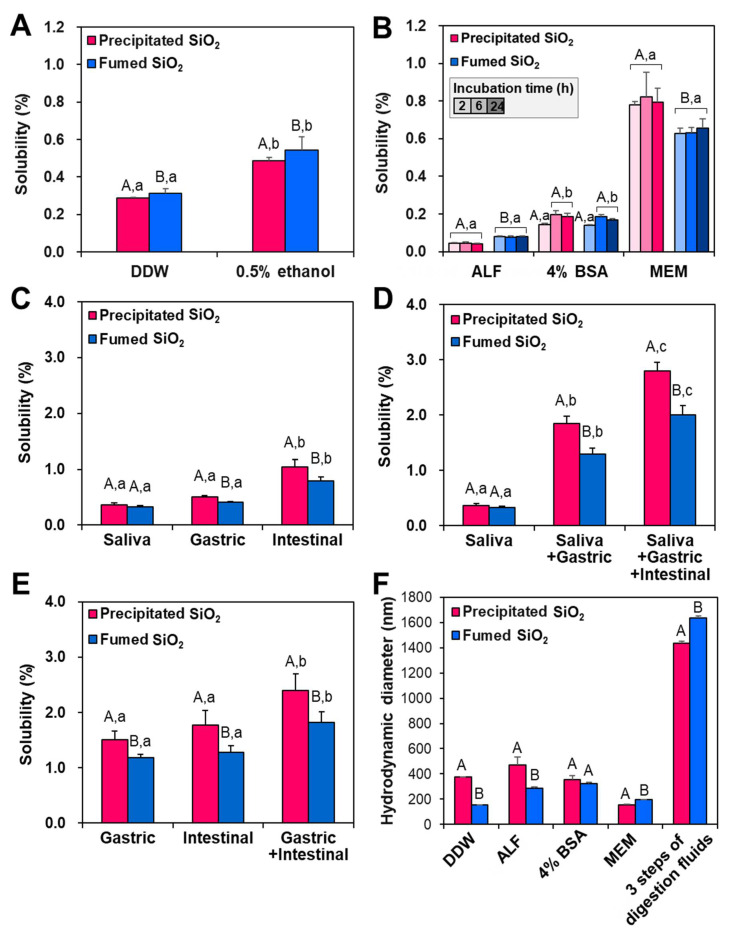
Dissolution properties of precipitated SiO_2_ and fumed SiO_2_ in (**A**) distilled and deionized water (DDW) and 0.5% ethanol; (**B**) artificial lysosomal fluid (ALF), 4% bovine serum albumin (BSA) solution, and minimum essential medium (MEM); (**C**,**D**) in vitro digestion fluids; and (**E**) ex vivo rat-extracted gastrointestinal (GI) fluids. (**F**) Hydrodynamic diameters of precipitated SiO_2_ and fumed SiO_2_ in DDW, ALF, 4% BSA solution, MEM, and three consecutive steps of in vitro digestion fluids. Different uppercase letters (A,B) indicate significant differences between precipitated SiO_2_ and fumed SiO_2_ under the same conditions (*p* < 0.05). Different lowercase letters (a,b,c) indicate significant differences between experimental conditions (*p* < 0.05).

The dissolution properties of both types of SiO_2_ particles were evaluated in distilled and deionized water (DDW) and 0.5% ethanol solution according to Organization for Economic Co-operation and Development (OECD) test guideline (TG) 105 [30]. The solubility of precipitated and fumed SiO_2_ particles in DDW was 0.29 and 0.31%, respectively, and slightly increased in 0.5% ethanol solution (0.49 and 0.54% for the former and the latter, respectively) (Figure 2A). Extremely low dissolutions (< 0.1%) of both particles were found in artificial lysosomal fluid (ALF), with the same tendency obtained in DDW and ethanol, and the solubility did not increase even after 24 h (Figure 2B). The solubility of fumed SiO_2_ was significantly higher than that of precipitated SiO_2_ in DDW (Figure 2A), 0.5% ethanol solution (Figure 2A), and ALF (Figure 2B). On the other hand, the solubility of both particles was ~0.19% at 6–24 h in 4% bovine serum albumin (BSA) solution, without significant differences between the two types of particles (*p* > 0.05) (Figure 2B). The solubility of precipitated SiO_2_ and fumed SiO_2_ increased to ~0.81 and ~0.65%, respectively, in minimum essential medium (MEM), showing significantly higher solubility for the former than the latter (*p* < 0.05) (Figure 2B). Low solubility of SiO_2_ particles was also measured in in vitro digestion fluids—such as saliva, gastric fluids, and intestinal fluids—and the highest solubility was found in the intestinal fluid, compared with saliva or gastric fluid (Figure 2C). When three consecutive steps of digestion fluids, such as saliva followed by gastric and intestinal fluids, were applied, the solubility of precipitated SiO_2_ and fumed SiO_2_ was 2.8 and 2.0%, respectively, with significant difference (*p* < 0.05) (Figure 2D). The same tendency was also obtained using ex vivo rat-extracted gastrointestinal (GI) fluids, showing 2.4 and 1.8% solubility for precipitated SiO_2_ and fumed SiO_2_, respectively (Figure 2E).

The hydrodynamic diameters of SiO_2_ particles were also checked in DDW, ALF, 4% BSA solution, MEM, and three consecutive steps of in vitro digestion solutions, where their solubility was evaluated. Figure 2F shows that the hydrodynamic diameters of precipitated SiO_2_ were significantly larger than those of fumed SiO_2_ in DDW and ALF, whereas fumed SiO_2_ had statistically larger hydrodynamic diameters than precipitated SiO_2_ in MEM and the three consecutive steps of digestion solutions. No statistical difference in the hydrodynamic diameters between the two types of particles was found in 4% BSA solution (*p* > 0.05). The smallest and largest hydrodynamic diameters of SiO_2_ particles were measured in MEM and the three steps of digestion fluids, respectively.

### 2.4. Optimization of CPE for SiO_2_

**Figure 3:**
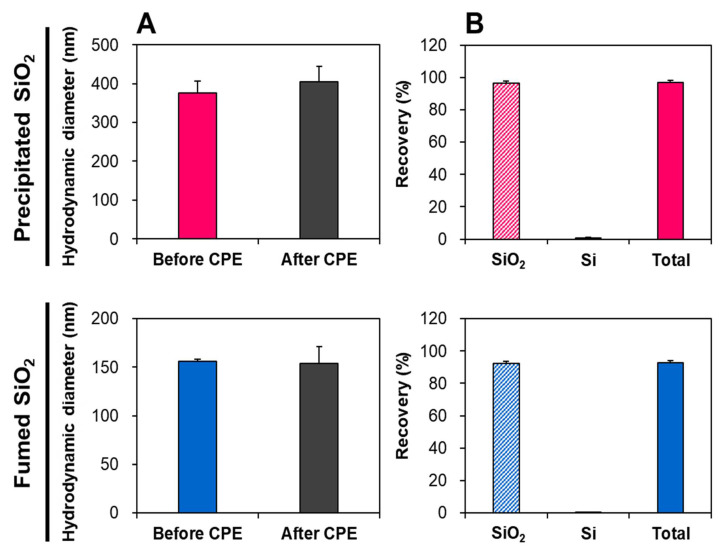
(**A**) Hydrodynamic diameters of food additive SiO_2_ before and after cloud point extraction (CPE). (**B**) Recoveries (%) of SiO_2_ particles, Si ions, and total Si levels, obtained via CPE. No significant differences between before and after CPE were found (*p* > 0.05).

The CPE for two differently manufactured types of SiO_2_ particles was optimized using TX-114 at pH 3.0. The micelle formation of particles in TX-114 easily occurs at the IEP, but a slightly greater pH than the IEPs (1.53 and 1.92 for precipitated SiO_2_ and fumed SiO_2_, respectively, Figure S1) was chosen. Indeed, the addition of sodium chloride (NaCl) during the CPE process at a pH higher than the IEP elevates the zeta potential values of NPs [31], which reduces the electrostatic repulsion between negatively charged NPs in TX-114-based micelles, thereby resulting in a net zero interaction and enhancing phase separation [31,32]. Hence, the particles as intact forms are precipitated in TX-114-based micelles, and Si ions released from the particles are present in the supernatants after centrifugation. As shown in Figure 3A, the hydrodynamic diameters of two differently manufactured SiO_2_ types in TX-114-rich precipitates obtained via CPE were similar to those of pristine SiO_2_, without significant differences (*p* > 0.05). This result was obtained after the digestion of organic matrices with HNO_3_ and 1 mL of hydrogen peroxide (H_2_O_2_). The ICP-AES analysis shows that the recoveries (%) as intact particle forms were 96.3 and 92.2% for precipitated SiO_2_ and fumed SiO_2_, respectively (Figure 3B). Meanwhile, the concentrations of Si ions released from the particles during the CPE procedure, found in the supernatants, were extremely low, showing 0.9 and 0.6% for precipitated SiO_2_ and fumed SiO_2_, respectively. Total detected Si levels in the precipitates and supernatants by CPE were 96.9 and 92.8% for precipitated SiO_2_ and fumed SiO_2_, respectively.

**Figure 4:**
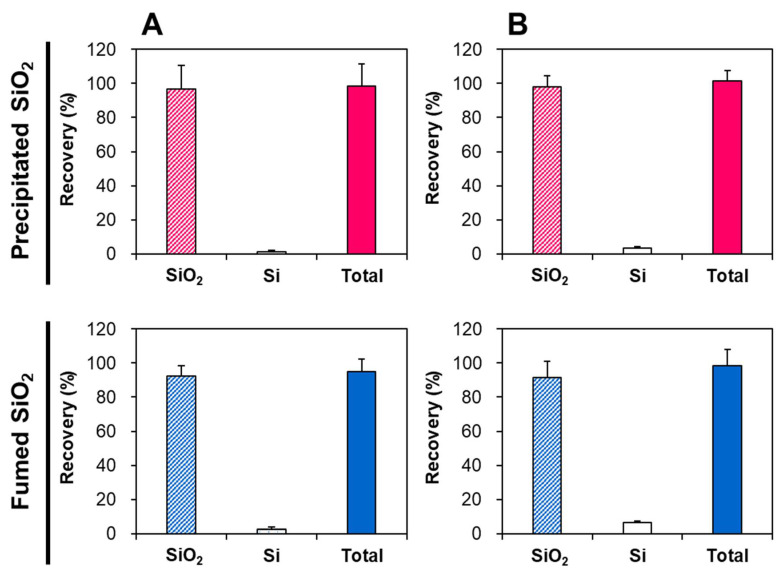
Recoveries (%) of SiO_2_ particles, Si ions, and total Si levels in (**A**) SiO_2_-spiked Caco-2 cells and (**B**) SiO_2_-spiked liver, obtained via cloud point extraction (CPE).

The CPE was also optimized with SiO_2_-spiked Caco-2 cells or liver tissue. In this case, we focused on the effect of ultrasonic homogenization—which was used to lyse the cells or tissues—on the inert size distribution of NPs. Figure 4 demonstrates that the recoveries (%) of both SiO_2_ particle forms, obtained via CPE in Caco-2 cells or liver tissue, ranged from 91.6 to 97.8%, although 1.5–6.7% of Si ions were found to be released from the particles after the CPE and lysis procedure. Slightly higher Si releases from the particles in SiO_2_-spiked liver cells than those in SiO_2_-spiked Caco-2 cells were also observed after CPE and lysis.

### 2.5. Intracellular Uptake and Intestinal Transport Fates of SiO_2_ Particles

**Figure 5:**
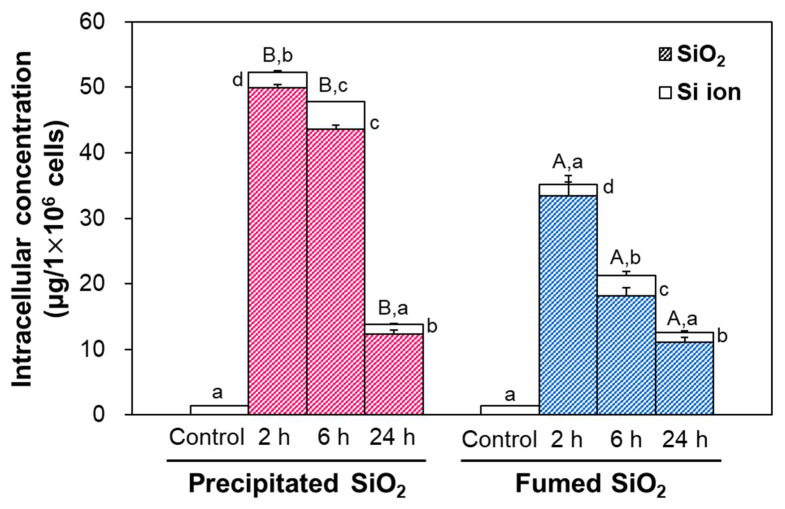
Intracellular uptakes and fates of SiO_2_ in Caco-2 cells, obtained via cloud point extraction (CPE). Different uppercase letters (A,B) indicate significant differences in total intracellular Si levels between precipitated SiO_2_ and fumed SiO_2_ (*p* < 0.05). Different lowercase letters (a,b,c,d) indicate significant differences between different incubation times (*p* < 0.05).

The uptake and intracellular fates of two differently manufactured types of SiO_2_ particles were evaluated in Caco-2 cells after incubation for 2, 6, and 24 h, and determined by applying the optimized CPE. The highest uptake amounts of the two differently manufactured SiO_2_ particle types were detected at 2 h post-incubation, and decreased with incubation time (Figure 5). Significant differences in uptake amount between precipitated SiO_2_ and fumed SiO_2_ were found, showing higher uptake of the former than the latter (*p* < 0.05). SiO_2_ particles inside cells were determined via CPE to be primarily present in particle form, regardless of incubation time or manufactured type. About 95% of both types of SiO_2_ was found in particle form at 2 h, and this figure decreased to ~88% at 24 h, without significant differences between the manufactured types (*p* > 0.05), whereas the proportion of Si ions increased as incubation time increased.

**Figure 6:**
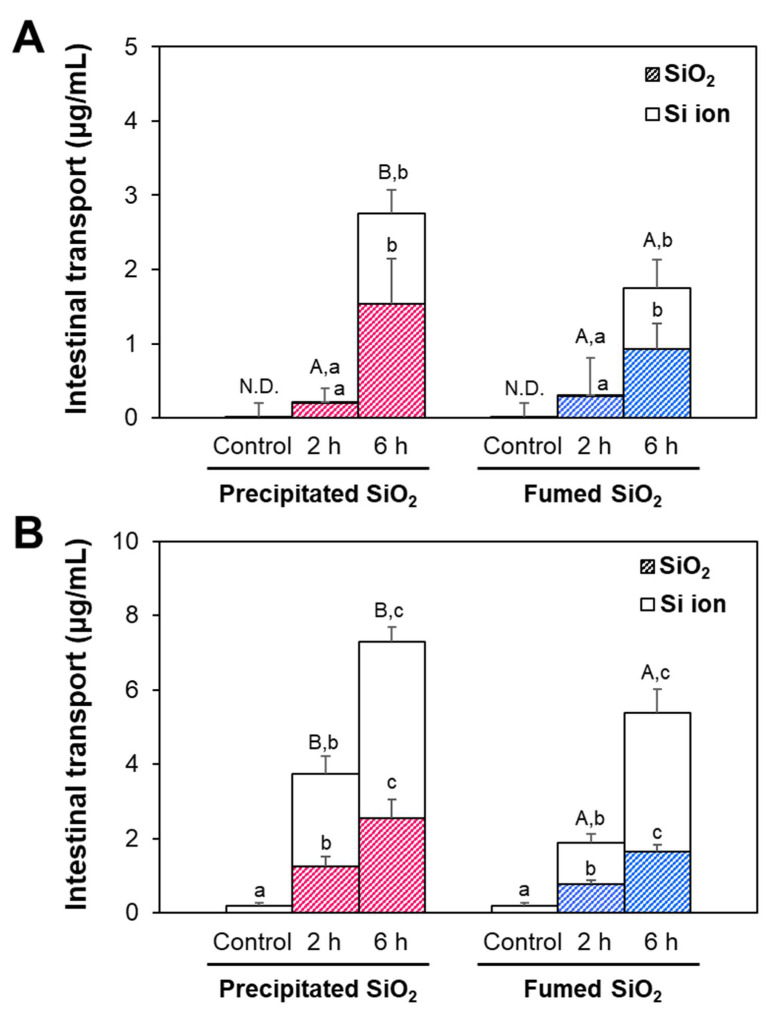
Intestinal transports and fates of SiO_2_ using (**A**) in vitro Caco-2 monolayer and (**B**) follicle-associated epithelium (FAE) models, obtained via cloud point extraction (CPE). Different uppercase letters (A,B) indicate significant differences in total Si transport levels between precipitated SiO_2_ and fumed SiO_2_ (*p* < 0.05). Different lowercase letters (a,b,c) indicate significant differences between different incubation times (*p* < 0.05). ND: not detectable.

The intestinal transports and fates of SiO_2_ particles were evaluated using in vitro 2D Caco-2 monolayer and 3D follicle-associated epithelial (FAE) models. Caco-2 monolayer and FAE models represent the intestinal tight junction barrier and microfold (M) cells in Peyer’s patches, respectively. The permeability of Lucifer yellow—a fluorescent dye that is only transported paracelluarly—was 2.3 and 2.4% in Caco-2 monolayer and FAE models, respectively, indicating well-established intestinal barriers [33,34]. The results show that the two types of particles were determined to be transported through both Caco-2 monolayer and M cells, but more massive transports by M cells were found (Figure 6). Total transported amounts of both kinds of SiO_2_ particles increased as incubation time increased, and significantly higher transport of precipitated SiO_2_ compared with fumed SiO_2_ was found in both models. Transport amounts (%) of precipitated SiO_2_ and fumed SiO_2_ at 6 h were ~1.0 and ~0.7% by Caco-2 monolayers, and ~2.1 and ~1.5% by the FAE model, respectively. It is worth noting that the recoveries (%) of SiO_2_ particles in both apical and basolateral sides ranged from 98.7 to 101.6% in all cases, supporting the accuracy of the analyzed transport levels (Table S1).

When the fate of SiO_2_ was determined by CPE, most SiO_2_ particles were present as particle forms at 2 h, and the portion of Si ions increased up to ~49% at 6 h in a Caco-2 monolayer model, regardless of manufactured type. On the other hand, ~65% of Si ions from precipitated SiO_2_ and fumed SiO_2_ were found at 2 and 6 h in an FAE model.

### 2.6. Oral Distribution and Fates of SiO_2_ Particles in Tissues

**Figure 7:**
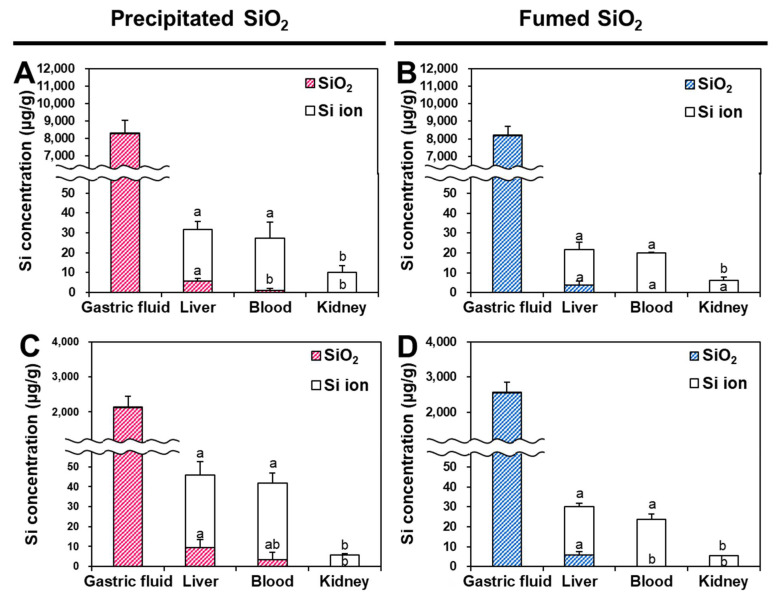
Tissue distribution and fates of (**A**,**C**) precipitated SiO_2_ and (**B**,**D**) fumed SiO_2_ in rats after single-dose oral administration of (**A**,**B)** 300 mg/kg or (**C**,**D**) 2000 mg/kg, obtained via cloud point extraction (CPE). Different lowercase letters (a,b) indicate significant differences between tissues (liver, blood, and kidney) (*p* < 0.05).

The distribution and fates of SiO_2_ particles were evaluated after a single-dose oral administration (300 or 2000 mg/kg) in rats. The doses were chosen based on the previous report, showing that the NOAEL of SiO_2_ NPs was more than 2000 mg/kg [18]. Two different doses (300 and 2000 mg/kg) were used to investigate whether the dose affects the oral particle/ionic forms of SiO_2_ particles. The timepoints for tissue distribution after oral administration of 300 or 2000 mg/kg were set at 2 and 10 h, based on our previous research [35,36]. The CPE model developed was applied to the gastric fluid, liver, blood, and kidneys after oral administration in rats, and Si levels were analyzed in the precipitates and supernatants via CPE. As shown in Figure 7, extremely low amounts (less than 0.2%) of SiO_2_ particles were dissolved in the gastric fluid, with no significant differences between administered doses or manufactured type (*p* > 0.05). When SiO_2_ particles were distributed in the liver through intestinal and hepatic portal vein transport, the majority of the fates (~81%) of precipitated SiO_2_ and fumed SiO_2_ were ionic forms, regardless of manufactured type. Moreover, particulate forms were detected in the liver in a dose-dependent manner, based on more particle forms being detected at 2000 mg/kg than at 300 mg/kg. When SiO_2_ particles were absorbed into the bloodstream, greater proportions of both types of SiO_2_ were present as ionic forms, and then completely dissolved into Si ions in the kidneys. A slightly greater proportion of particulate forms of precipitated SiO_2_ compared with fumed SiO_2_ was found in the blood. Almost 100% of fumed SiO_2_ was present in ionic forms after entering systemic circulation and distribution in the kidneys.

## 3. Discussion

The biological fate and toxicity of NPs can differ according to their physicochemical properties, which are mainly related to different manufacturing methods. The results of physicochemical characterization of the most widely applied food additive SiO_2_ particles—precipitated, and fumed forms—demonstrate different characteristics; the constituent particle sizes of precipitated SiO_2_ and fumed SiO_2_ were similar, with no significant difference (*p* > 0.05, Figure 1), but present as aggregates, as shown in the SEM/TEM images (Figure 1) and DLS results (Table 1). The hydrodynamic diameters of fumed SiO_2_ in DW were much smaller than those of precipitated SiO_2_, as indicated by the high proportion of the fraction less than 100 nm and small Z-average size, contributing to high BET surface area for fumed SiO_2_ (Table 1). The zeta potential values of both particles were negative, but slightly more negative charge was found for precipitated SiO_2_ compared with fumed SiO_2_, leading to a lower IEP for the former (pH 1.53) than for the latter (pH 1.92) (Figure S1).

The solubility of SiO_2_ particles differed based on their manufacturing method and fluid type. Fumed SiO_2_ had significantly higher dissolution properties in DDW, 0.5% ethanol, and ALF than did precipitated SiO_2_, and the solubility increased in the order of ALF < 4% BSA solution < DDW < 0.5% ethanol < MEM < digestion fluids, in all cases (Figure 2). On the other hand, the solubility of precipitated SiO_2_ was higher than that of fumed SiO_2_ in MEM, in vitro digestion fluids, and ex vivo rat-extracted GI fluids (Figure 2B–E). This discrepancy can be explained by the DLS results obtained in each fluid tested, showing larger hydrodynamic diameters of precipitated SiO_2_ in DDW and ALF, but smaller diameters in MEM and the three steps of digestion solutions, compared to fumed SiO_2_ (Figure 2F). Hence, aggregation of SiO_2_ under test conditions can result in low solubility, related to its small specific surface area and low reactivity. The different solubility depending on fluid type can be explained by several factors; interaction between SiO_2_ and various components of MEM—such as 10% fetal bovine serum (FBS), amino acids, vitamins, and salts—can increase the solubility to some extent, as observed by high dissolution in MEM (Figure 2B). Indeed, the role of cell culture medium as a dispersant of NPs has been well reported [37,38], which is consistent with the smallest hydrodynamic diameters of SiO_2_ particles being observed in MEM (Figure 2F). The effect of 4% BSA—which reflects albumin level in the plasma [39]—on solubility seems to be minor when the solubility between DDW and 4% BSA solution is compared (Figure 2A,B). It is interesting to note that the highest solubility of SiO_2_ particles was found in the three consecutive steps of digestion fluids, where their high aggregation was found (Figure 2D–F); this result may be related to the effect of pH, as demonstrated by the high aggregation of SiO_2_ particles at acidic pH due to their IEPs at low pH (Figure S1A). It was demonstrated that electrostatic repulsive forces between SiO_2_ particles are minimized, whereas van der Waals attractive forces increase, at the IEP—inducing aggregation [40], which is also seen in Figure S1B. High dissolution properties of SiO_2_ under alkaline conditions, as observed in intestinal fluid (Figure 2C), can contribute to its high solubility in three steps of digestion fluids, where duodenal and bile conditions are reflected (~pH 8.2, Table S3) [41]. Meanwhile, the higher solubility of precipitated SiO_2_ particles than of fumed SiO_2_ particles is in good agreement with the report demonstrating that precipitated SiO_2_ has more silanol groups (Si-OH) per unit surface than fumed SiO_2_, resulting in high solubility [26]. It is worth noting that the solubility in an in vitro three consecutive steps of digestion model (Figure 2D) was highly correlated with ex vivo results obtained using rat-extracted GI fluids, showing about 2.4 and 1.8% solubility for precipitated SiO_2_ and fumed SiO_2_, respectively (Figure E). These results suggest that most SiO_2_ particles are not dissolved, and present as particle forms under GI conditions.

All validation parameters of quantitative analytical methods for SiO_2_ particles in biomatrices—such as linearity, low RMSE, high recovery (%), low repeatability (RSD %), and sensitive LOD and LOQ values—reveal the accuracy and precision of the quantitative methods, including digestion procedure and ICP-AES analysis (Table 2). This is also supported by the high recoveries (93–102%) of total Si levels from Si ions and SiO_2_ particles obtained via CPE (Figure 3 and Figure 4). The reliability of the CPE to detect intact SiO_2_ was confirmed by the statistically similar hydrodynamic diameters of pristine SiO_2_ before and after CPE (Figure 3A), and by the high recoveries (92–96%) as particle forms in TX-114-based precipitates obtained via CPE (Figure 3B). The recoveries of SiO_2_ particles in the precipitates obtained by CPE ranged from 92 to 98% in SiO_2_-spiked biomatrices (Caco-2 cells and rat liver) (Figure 4), supporting the efficacy of the CPE method. Slightly increased levels of Si ions (3.7–6.7%) were detected in SiO_2_-spiked liver (Figure 4B), probably resulting from the ultrasonic homogenization process for tissue lysis prior to CPE application. Hence, the tissue fates of SiO_2_ after oral administration were evaluated considering the proportions of Si ions versus SiO_2_ in SiO_2_-spiked liver, obtained via CPE (Figure 4B).

The intracellular fates of precipitated SiO_2_ and fumed SiO_2_ in Caco-2 cells, determined by CPE, were mainly particle forms, even after 24 h of incubation, but ~95% of particle forms at 2 h decreased to ~89% at 24 h, without significant differences between the manufactured types (*p* > 0.05, Figure 5), suggesting slow decomposition of SiO_2_ inside cells. The uptake amounts of both particles decreased as incubation time increased, implying possible exocytosis or excretion of the decomposed particles. Meanwhile, the uptake amounts of precipitated SiO_2_ were significantly higher than those of fumed SiO_2_ at 2–24 h, which seems to be associated with the smaller hydrodynamic diameters of the former than the latter in the three steps of in vitro digestion solutions (Figure 2F). Indeed, intracellular organelles such as endosomes and lysosomes have acidic pH and many enzymes for the degradation of molecules [42,43]. This result suggests that small particles can be more easily and massively taken up by cells [44]. Indeed, energy-dependent endocytosis of SiO_2_ particles has been well reported [45]. The intestinal transport amounts of precipitated SiO_2_ through the Caco-2 monolayer and M cells were also significantly higher than those of fumed SiO_2_ (Figure 6), indicating efficient intestinal transport of SiO_2_ with small hydrodynamic diameters. Total transport (%) of precipitated SiO_2_ and fumed SiO_2_ by both the Caco-2 monolayer and FAE models at 6 h was ~3.0 and ~2.2%, respectively (Table S1), which is in good agreement with our in vivo oral absorption [46]. It should be noted that energy-dependent transport of SiO_2_ particles through intestinal barriers has been demonstrated [8,46]. However, intestinal transport fates of SiO_2_ were different depending on the types of intestinal transport models. The two differently manufactured particle types were mainly present as particles at 2 h, and ~51% particle forms were found at 6 h in a Caco-2 monolayer model (Figure 6A), indicating slow dissolution during transportation through intestinal tight junction barriers. Conversely, about 63% and 67% of Si ions were detected at 2 and 6 h, respectively, in the FAE model (Figure 6B), implying relatively fast dissolution of SiO_2_ during intestinal transport via M cells. This may be related to the role of lymphocyte Raji B cells in the FAE model, which are involved in the immune system [47].

After oral administration of two SiO_2_ particles in rats, significantly higher amounts of precipitated SiO_2_ were found in all organs analyzed, compared with those of fumed SiO_2_ (Figure 7), which is consistent with the cellular uptake (Figure 5) and intestinal transport (Figure 6) amounts. Both types of particles were not dissolved in the gastric fluid, but ~81% of ionized forms were detected in the liver regardless of administered doses, indicating that the majority of the oral fates of both SiO_2_ types were ionic forms in the liver. On the other hand, about 4 and 7% particle forms of precipitated SiO_2_ were found in the blood at 300 and 2000 mg/kg (Figure 7A–C), respectively, whereas less than 1% of particles remained intact in the blood after the oral administration of fumed SiO_2_, regardless of the administered dose (Figure 7B–D). No particle forms were detected in the kidneys in all cases. Taken together, SiO_2_ particles were present as intact particle forms in the stomach, and slowly dissolved into Si ions during intestinal cellular uptake and transport. They were then more decomposed after distribution in the liver and entering the circulation system, and finally excreted as Si ions in the kidneys. Indeed, SiO_2_ undergoes hydrolysis to form silicic acid, and in vivo biodegradation of SiO_2_ to silicic acid was demonstrated [20,36]. The metabolic pathway involved in the degradation or decomposition of SiO_2_ particles in the tissues and cells remains to be elucidated in the near future.

## 4. Materials and Methods

### 4.1. Materials

Food additive precipitated SiO_2_ (SIPERNAT 22S) and fumed SiO_2_ (AEROSIL 200F) particles were purchased from Evonik Industries AG (Essen, Germany). Stock solutions (1 mg/mL) of SiO_2_ particles were prepared in DW, stirred for 30 min, sonicated for 15 min, and then diluted (100 μg/mL) just prior to all experiments.

TX-114, calcium chloride dihydrate (CaCl_2_·2H_2_O), formaldehyde, citric acid, glycerin, sodium citrate dihydrate, sodium tartrate dihydrate, sodium lactate, sodium pyruvate, glycine, potassium thiocyanate (KSCN), sodium bicarbonate (NaHCO_3_), urea, α-amylase, uric acid, mucin, D-(+)-glucose, glucuronic acid, glucosamine hydrochloride, BSA, pepsin, pancreatin, lipase, bile, Lucifer yellow CH, and Si standard solution were provided by Sigma-Aldrich (St. Louis, MO, USA). Nitric acid (HNO_3_), hydrogen peroxide (H_2_O_2_), hydrogen fluoride (HF), sodium chloride (NaCl), sodium hydroxide (NaOH), sodium phosphate (NaHPO_4_), potassium chloride (KCl), potassium dihydrogen phosphate (KH_2_PO_4_), ethyl alcohol, and hydrochloride (HCl) were supplied by Samchun Pure Chemical Co., Ltd. (Pyeongtaek, Gyeonggi-do, Korea). Sodium sulfate (Na_2_SO_4_), magnesium chloride hexahydrate (MgCl_2_·6H2O_)_, and sodium dihydrogen phosphate dihydrate (NaH_2_PO_4_·2H_2_O) were acquired from Junsei Chemical Co., Ltd. (Tokyo, Japan). Conical-bottomed glass centrifuge tubes (15 mL) were obtained from Daeyoung Science (Seoul, Korea). MEM, Roswell Park Memorial Institute (RPMI) 1640 medium, Dulbecco’s modified Eagle’s medium (DMEM), heat-inactivated FBS, penicillin, streptomycin, Hanks’ balanced salt solution (HBSS), and Dulbecco’s phosphate-buffered saline (DPBS) were purchased from Welgene Inc. (Gyeongsangbuk-do, Korea). Matrigel^®^ and Transwell^®^ polycarbonate inserts were supplied by Corning Inc. (Corning, NY, USA) and SPL Life Science Co., Ltd. (Pocheon, Gyeonggi-do, Korea), respectively.

### 4.2. Cell Culture

Human intestinal epithelial Caco-2 cells and non-adherent human Burkitt lymphoma Raji B cells were supplied by Korean Cell Line Bank (Seoul, Korea). Caco-2 cells and Raji B cells were cultured in MEM and RPMI 1640 medium, respectively, containing 10% FBS, 100 units/mL of penicillin, and 100 μg/mL of streptomycin under 5% CO_2_ atmosphere at 37 °C.

### 4.3. Animals

Seven-week-old female Sprague Dawley (SD) rats weighing around 130–170 g were purchased from Koatech Co. (Pyeongtaek, Gyeonggi-do, Korea). The animals were housed in plastic laboratory animal cages in a ventilated room, maintained at 20 ± 2 °C and 60% ± 10% relative humidity with a 12 h light/dark cycle. Water and commercial laboratory complete food for rats were available ad libitum. The animals were environmentally acclimated for 7 days before experimental treatments. All animal experiments were performed in accordance with the guidelines established by the Animal and Ethics Review Committee of Seoul Women’s University (SWU IACUC-2020A-8).

### 4.4. Characterization

Constituent particle sizes and morphologies of SiO_2_ particles were determined via field emission (FE)-SEM (JSM-7100F, JEOL, Tokyo, Japan) and high-resolution TEM (JEM-2100F, JEOL). For FE-SEM analysis, the samples were prepared by dropping 20 μL of SiO_2_ stock solutions (0.1 mg/mL) onto a mount (Specimen Mount, JEOL, Tokyo, Japan) with a carbon tape (5 mm × 5 mm; E-SONG EMC, Seoul, Korea), and then dried at room temperature. The sample surface was then coated with Pt/Pd via a sputtering process for 30 s. FE-SEM images were obtained at an acceleration voltage of 15 kV. For TEM analysis, the suspensions (0.1 mg/mL) of SiO_2_ particles were prepared in ethanol and sonicated for 15 min. Then, 5 μL of the suspensions were dropped on a 200-mesh, carbon-coated copper grid (PELCO^®^ TEM Grids, Ted Pella Inc., Redding, CA, USA) and dried at room temperature. TEM images were obtained at an accelerating voltage of 200 kV. The average particle sizes and size distributions of SiO_2_ particles in the FE-SEM and TEM images were analyzed using ImageJ software (version 1.53a, National Institutes of Health, Bethesda, MD, USA).

Hydrodynamic diameters and zeta potentials of SiO_2_ particles were measured via DLS and electrophoretic light scattering (ELS), respectively, using a Zetasizer Nano system (Malvern Instruments, Worcestershire, UK). For DLS and ELS analysis, 1 mL of SiO_2_ stock suspensions (1 mg/mL) was placed in disposable plastic cuvettes and measured at room temperature.

The specific surface area was determined via the BET method, using N_2_ gas adsorption–desorption isotherms (at 77 K) of the particles. The moisture in the particles was removed by drying at 100 °C for 2 h prior to analysis, and then measured with a surface area analyzer (TriStar II 3020, Micromeritics, Norcross, GA, USA).

### 4.5. Digestion of Organic Matrices, Microwave Digestion, and ICP-AES Analysis

SiO_2_ particles were quantified by measuring total Si contents using ICP-AES (JY2000 Ultrace, HORIBA Jobin Yvon, Longjumeau, France), with Si standard solutions of different concentrations. To digest organic materials in the supernatants after solubility or CPE experiments, digestion was performed with 10 mL of ultrapure HNO_3_ and 1 mL of H_2_O_2_ at 180 °C until the solution was colorless and completely evaporated. The same digestion procedure was applied to measure the hydrodynamic diameters of SiO_2_ in the precipitates via CPE. For quantitative analysis of the precipitates containing SiO_2_ via CPE, the samples were transferred to perfluoroalkoxy microwave digestion vessels, and 6 mL of 70% HNO_3_ and 1 mL of 40% HF were added. The samples were digested for 55 min at 1600 W in a microwave digestion system (ETHOS EASY, Milestone Srl, Sorisole, Italy), by irradiation at 120, 160, and 210 °C for 15, 10, and 30 min, respectively, followed by holding for 1 min. After digestion, all samples were diluted to appropriate volumes with DDW, shaken manually, and analyzed via ICP-AES with a radiofrequency power of 1000 W and an argon flow rate of 0.02 mL/min in the nebulizer. Method blanks were determined by performing the same procedure in the absence of Si samples.

### 4.6. Validation of Quantitative Analytical Method

The method for quantitative analysis was validated by evaluating the LOD, LOQ, linearity, recovery (%), accuracy, precision, and matrix effects according to the International Conference on Harmonization (ICH, Q2B) guidance [48]. All validation parameters were evaluated with Si standard solution, precipitated and fumed SiO_2_, and SiO_2_-spiked matrices (cells and liver), at SiO_2_ concentrations of 0, 2.5, 5, 25, and 50 μg/g (corresponding to Si concentrations of 0, 1.2, 2.3, 12, and 23 μg/g, respectively). The LOD and LOQ were calculated according to the following equations: LOD = 3.3 × σ/S; and LOQ = 10 × σ/S (σ: standard deviation of the response; S: slope of the calibration curve). The linearity of the quantification was evaluated as a coefficient of determination values (R^2^). The accuracy was evaluated by the recovery (%) of known added SiO_2_ at different concentrations. The precision was determined by repeatability and expressed as RSD% from measurements of five replicates [49]. Uncertainty of the obtained calibration curves was validated by calculating RMSE values [50].

### 4.7. In Vitro Dissolution Properties of SiO_2_

The solubility of SiO_2_ particles in water was evaluated according to the flask method of OECD TG 105 water solubility [30]. SiO_2_ particles (50 g/L) were dispersed in DDW and preincubated with shaking at 30 °C for 24 h to achieve the saturation equilibrium, followed by shaking at 20 °C for 24, 48, and 72 h. The supernatants were then collected via ultracentrifugation (16,000× *g*) for 15 min, filtered through a syringe filter (pore size 0.22 μm), and quantitative analysis of the dissolved Si from SiO_2_ was performed using ICP-AES (JY2000 Ultrace, HORIBA Jobin Yvon). If the differences in Si concentrations measured in the samples at 20 °C for 24, 48, and 72 h were less than 15%, the concentrations were used for the solubility values.

The solubility of SiO_2_ was investigated in ALF, the composition of which is summarized in Table S2 [51]. SiO_2_ particles (1 mg/mL) were dispersed in ALF and incubated for designated times (2, 6, and 24 h) at 37 °C. The solubility of SiO_2_ was also assessed in the presence of 4% BSA and MEM under the same conditions. After incubation, the samples were centrifuged at 16,000× *g* for 15 min, and the supernatants were subjected to ICP-AES analysis.

The solubility of SiO_2_ was also evaluated in an in vitro three steps of digestion model consisting of simulated saliva, gastric fluids, and intestinal fluids, as described by Peters et al. (Table S3) [9]. All simulated digestion fluids were prepared on the day of the experiment, and the fluids were preheated to 37 °C for at least 2 h. For dissolution experiments in each digestion fluid, SiO_2_ particles (1 mg/mL) were dispersed in simulated saliva, gastric fluids, and intestinal fluids, and incubated for 5 min, 2 h, and 2 h, respectively, on a head-over-head rotator at 37 °C. For consecutive digestion experiments, SiO_2_ particles (1 mg/mL) were dispersed in simulated saliva (6 mL) for 5 min at 37 °C, followed by sequential gastric digestion for 2 h at 37 °C by the addition of gastric fluid (12 mL). Then, intestinal digestion was further performed for 2 h at 37 °C by the addition of duodenal (12 mL) and bile (6 mL) fluids to the suspensions. After each digestion step, the samples were centrifuged at 16,000× *g* for 15 min, and the Si contents in the supernatants were measured using ICP-AES (JY2000 Ultrace, HORIBA Jobin Yvon).

### 4.8. Ex Vivo Dissolution Properties of SiO_2_ in Rat-Extracted GI Fluids

Rat gastric and intestinal fluids were prepared as described by Lee et al. [52]. Briefly, the stomachs and small intestines of rats were collected and washed with saline, and then the gastric and intestinal fluids were extracted. The supernatants of the gastric and intestinal fluids were obtained by centrifugation at 16,000× *g* for 15 min at 4 °C, and were used for ex vivo solubility experiments. Ex vivo digestion was performed in the same manner as described in the in vitro three steps of digestion model (2:3 *v*/*v* gastric:intestinal). For solubility in each digestion fluid, SiO_2_ particles (1 mg/mL) were dispersed in the rat-extracted gastric and intestinal fluids, respectively, and incubated for 2 h at 37 °C. For consecutive digestion experiments, SiO_2_ particles were dispersed in the gastric fluid (0.5 mL) at a concentration of 1 mg/mL and incubated for 2 h at 37 °C. The suspensions were then combined with the intestinal fluid (0.75 mL) and incubated for a further 2 h at 37 °C. After each digestion step, the samples were centrifuged at 16,000× *g* for 15 min, and Si concentrations in the supernatants were analyzed using ICP-AES (JY2000 Ultrace, HORIBA Jobin Yvon).

### 4.9. Optimization of CPE for SiO_2_

The CPE method for SiO_2_ was established with the stock solutions of food additive SiO_2_ (100 μg/mL), in the absence of matrices. The SiO_2_ solutions (7 mL) were added to conical-bottomed glass centrifuge tubes, and the pH was adjusted to 3.0 with diluted HNO_3_. Then, 0.5 mL of 5% (*w*/*v*) TX-114 and 0.75 mL of 0.2 M NaCl were added to the samples. After dilution to 10 mL with DDW, the mixtures were incubated for 30 min at 45 °C and then centrifuged for 5 min at 2500× *g* at 25 °C to facilitate phase separation. The precipitates of SiO_2_ particles in the TX-114-rich phase and the supernatants of the upper aqueous phase were collected for Si quantification via ICP-AES (JY2000 Ultrace, HORIBA Jobin Yvon). The recoveries (%) of SiO_2_ particles in intact particle forms in the precipitated TX-114-rich phase were checked.

The CPE method for SiO_2_ particles in cells was optimized using SiO_2_-spiked Caco-2 cells. Briefly, the cells (1 × 10^6^ cells) were resuspended in 1 mL of DDW and transferred to conical-bottomed glass centrifuge tubes. Then, 1 mL of SiO_2_ stock solution (100 μg/mL) was added to the cell suspension. The prepared SiO_2_-spiked cells were sonicated four times for 10 s at 150 W on ice using an ultrasonic homogenizer (Sonics & Materials Inc., Newtown, CT, USA). After dilution to 7 mL with DDW, the same CPE procedure for food additive SiO_2_ was applied, as described above.

The CPE method for SiO_2_ particles in tissues was optimized using SiO_2_-spiked rat liver. The rat liver was collected and chopped with scissors, and 0.1 g of the chopped tissues was transferred to conical-bottomed glass centrifuge tubes. After adding 1 mL of SiO_2_ stock solution (100 μg/mL) to the chopped tissues, the volume was adjusted to 7 mL with DDW. The SiO_2_-spiked tissues were homogenized on ice using an ultrasound homogenizer (Sonics & Materials Inc.), and the same CPE procedure applied to food additive SiO_2_ was performed, as described above.

### 4.10. Cellular Uptake and Intracellular Fates of SiO_2_

The cells were plated at a density of 1 × 10^6^ cells/well and incubated with SiO_2_ particles (500 μg/mL) for 2, 6, and 24 h. After incubation, the cells were washed three times with DPBS and harvested with a scraper. Then, the cells were centrifuged for 1 min at 3000 rpm at 4 °C, and resuspended in 1 mL of DDW to determine the intracellular fate of SiO_2_ via CPE. Briefly, the suspended cells (1 mL) were added to conical-bottomed glass centrifuge tubes and lysed as described in Section 4.9. Then, the volume was adjusted to 7 mL with DDW, and the same CPE procedure for SiO_2_ was applied (see Section 4.9). Cells in the absence of SiO_2_ particles were used as controls. Si concentrations in the precipitates and supernatants, as measured by CPE, were analyzed using ICP-AES (JY2000 Ultrace, HORIBA Jobin Yvon).

### 4.11. Intestinal Transport and Fates of SiO_2_

A Caco-2 monoculture model was used to evaluate the transport of particles through the intestinal epithelial tight junction barrier. After Matrigel^®^ diluted in serum-free DMEM was coated on a Transwell^®^ insert for 1 h, the supernatants were removed, and the inserts were washed with serum-free DMEM. Caco-2 cells (4.5 × 10^5^ cells/well) were seeded on the upper insert and grown for 21 days until the transepithelial electrical resistance (TEER) value reached more than 300 Ω cm^2^. Then, the apical medium of the monolayers was replaced by medium containing SiO_2_ particles (500 μg/mL), and incubation was continued for 2 and 6 h. The pH of the apical and basolateral media was 7.4.

The FAE model, mimicking the M cells of Peyer’s patches in the intestinal epithelium, was prepared as previously described [53,54]. Briefly, Caco-2 cells (1 × 10^6^ cells/well) were seeded on the upper insert and grown for 14 days. Raji B cells (1 × 10^6^ cells/well) in DMEM were added to the basolateral inserts and co-cultured for 5 days (150–200 Ω cm^2^). Then, the apical medium of the monolayers was replaced by medium containing SiO_2_ particles (500 μg/mL), and incubation was continued for 2 and 6 h. The CPE method was applied by adding 1 mL of apical and basolateral solutions to conical-bottomed glass centrifuge tubes and diluting to 7 mL with DDW. The same CPE procedure for SiO_2_ was applied (see Section 4.9). Si quantifications in the precipitates and supernatants, obtained via CPE, were performed using ICP-AES (JY2000 Ultrace, HORIBA Jobin Yvon).

The permeability of Lucifer yellow, a marker of tight junction integrity, was checked as described previously [55,56]. The apical and basolateral sides of the Caco-2 monolayer and FAE models were washed with HBSS, and then the apical media were replaced with Lucifer yellow solution (300 μg/mL in HBSS). After incubation for 1 h at 37 °C, the apical and basolateral media were collected, and fluorescence was measured with a fluorescence microplate reader at excitation and emission wavelengths of 430 nm and 540 nm, respectively. The permeability (%) was calculated according to the following formula:

Permeability (%)=(apical medium−blank)/(basolateral mediam−balnk)×100

### 4.12. In Vivo Oral Distribution and Fate Determination of SiO_2_ in Tissues

Two groups of eight-week-old female SD rats (*n* = 4 per group) received a single dose of 300 or 2000 mg/kg of SiO_2_ particles by oral gavage, and one additional group of three rats received an equivalent volume of DW as controls. One gender (female) was used based on our previous research, showing no effect of gender on tissue distribution [36]. Tissue samples of blood, gastric fluid, kidneys, and liver were collected at time to peak concentration (300 mg/kg, 2 h; 2000 mg/kg, 10 h) after euthanasia by CO_2_ [35,57]. The tissue samples were stored at −80 °C until analysis.

The kidney and liver tissues were chopped with scissors. Then, 0.1 g of the chopped tissues was transferred to conical-bottomed glass centrifuge tubes and homogenized (Sonics & Materials Inc.) on ice in 7 mL of DDW. The biofluids (0.1 g), such as blood and gastric fluid, were directly transferred to conical-bottomed glass centrifuge tubes, and the volume was adjusted to 7 mL with DDW. The same CPE procedure for food additive SiO_2_ was applied (see Section 4.9). Si levels in the precipitates and supernatants, as obtained via CPE, were analyzed by ICP-AES (JY2000 Ultrace, HORIBA Jobin Yvon).

### 4.13. Statistical Analysis

Results were presented as means ± standard deviations. One-way analysis of variance with Tukey’s test was performed using the SAS Ver.9.4 (SAS Institute Inc., Cary, NC, USA) to determine the significances of intergroup differences. Statistical significance was accepted for *p* values of <0.05.

## 5. Conclusions

The constituent particle sizes of precipitated SiO_2_ and fumed SiO_2_ particles were similar, but different levels of solubility were found depending on the hydrodynamic diameters and aggregate states in the biofluid type. TX-114-based CPE for SiO_2_ was developed and optimized in intestinal cells and rat liver for its fate determination. The results show that the majority of the fates of SiO_2_ inside intestinal cells were particle forms, but slowly decomposed into ions during intestinal transport through the Caco-2 monolayer and M cells, regardless of manufactured type. Most SiO_2_ particles remained intact in the stomach, but ionic forms were primarily found in the liver and blood, and completely dissolved into ions in the kidneys. These findings will provide crucial information for understanding and predicting the potential toxicity of food additive SiO_2_ after oral intake. Further study is required in order to elucidate the metabolic pathway involved in the degradation or decomposition of SiO_2_ particles in the tissues.

## Figures and Tables

**Table 1:** Particle fractions, hydrodynamic diameters, and specific surface areas ^1^ of precipitated SiO_2_ and fumed SiO_2_.

Samples	Fraction (Number %)			Fraction (Mass %)			Z-Average Diameter (nm)	BET (m^2^/g)
	<100 nm	100–200 nm	>200 nm	<100 nm	100–200 nm	>200 nm		
Precipitated SiO_2_	0 ± 0 ^a^	4 ± 1 ^a^	96 ± 1 ^a^	0 ± 0 ^a^	1 ± 0 ^a^	99 ± 0 ^a^	375 ± 1 ^a^	167 ± 1 ^a^
Fumed SiO_2_	43 ± 2 ^b^	54 ± 2 ^b^	3 ± 0 ^b^	15 ± 1 ^b^	62 ± 2 ^b^	23 ± 1 ^b^	156 ± 1 ^b^	177 ± 1 ^b^

^1^ Specific surface areas, as measured by the Brunauer–Emmett–Teller (BET) method. Different lowercase letters (a,b) indicate significant differences between precipitated SiO_2_ and fumed SiO_2_ (*p* < 0.05).

**Table 2:** Validation parameters of the quantitative analytical method for SiO_2_ particles.

Samples	Linearity (R^2^)	RMSE	Recovery (%)	Repeatability (RSD%)	LOD (μg/g of Matrix)	LOQ (μg/g of Matrix)
Si standard solution	0.9999	0.0363	96.1 ± 2.7	1.95	–	–
Precipitated SiO_2_						
Pristine	0.9999	0.3318	98.0 ± 6.4	1.54	–	–
Cell	0.9998	0.2451	99.2 ± 1.5	2.94	0.50	1.55
Liver	1.0000	0.1565	97.3 ± 0.8	3.55	0.36	1.08
Fumed SiO_2_						
Pristine	0.9999	0.3987	103.3 ± 6.4	5.12	–	–
Cell	0.9999	0.3015	99.8 ± 5.2	3.41	0.37	1.15
Liver	0.9997	0.4520	105.0 ± 4.5	3.39	0.52	1.58

RMSE: root-mean-square error; RSD: relative standard deviation; LOD: limit of detection; LOQ: limit of quantification.

## Data Availability

The data presented in this study are available in the article and Supplementary Materials.

## Supplementary Materials

The following are available online at https://www.mdpi.com/article/10.3390/ijms22137035/s1.
