# Supplementary material for: Determination of Two Differently Manufactured Silicon Dioxide Nanoparticles by Cloud Point Extraction Approach in Intestinal Cells, Intestinal Barriers and Tissues
Source: Int J Mol Sci. 2021 Jun 29;22(13):7035. doi: 10.3390/ijms22137035 (PMC8268481; doi:10.3390/ijms22137035)
Supplement: Supplementary file 1 [file ijms-22-07035-s001.zip › ijms-1232175-supplementary.pdf]

Determination of Two Differently Manufactured Silicon Dioxide Nanoparticles by Cloud Point Extraction Approach in Intestinal Cells, Intestinal Barriers, and Tissues

§Na-Kyung Yoo, §Ye-Rin Jeon, and Soo-Jin Choi *

Division of Applied Food System, Major of Food Science & Technology, Seoul Women's University, Seoul 01797, Republic of Korea; iko0105@swu.ac.kr (N.-K.Y.); yrjeon0715@swu.ac.kr (Y.-R.J.)

§ Equally contributed to the work reported

* Correspondence: sjchoi@swu.ac.kr; Tel.: +82-2-970-5634; Fax: +82-2-970-5977

Received: date; Accepted: date; Published: date

Supplementary Materials

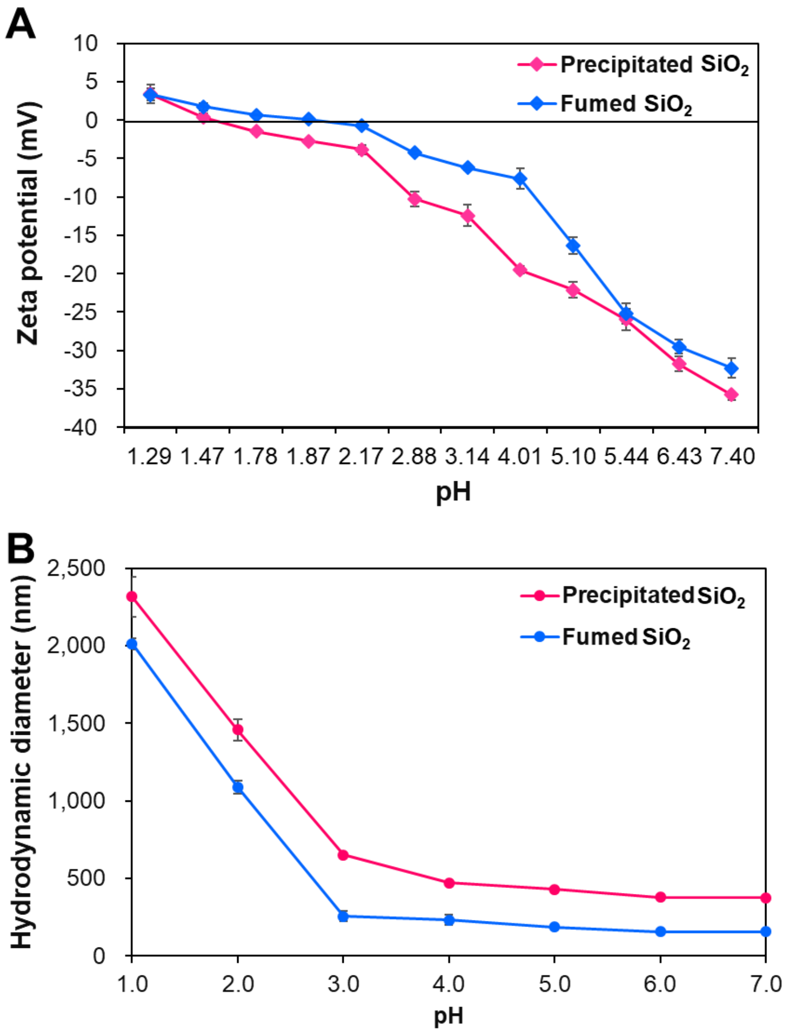

Figure S1. (**A**) Zeta potentials and (**B**) hydrodynamic diameters of precipitated SiO_2 and fumed SiO_2 suspension as a function of pH.

Table S1. Recoveries and transport amounts (%) of precipitated SiO_2 and fumed SiO_2 by Caco-2 monolayer and follicle-associated epithelium (FAE) models

		**Precipitated SiO_2**		**Fumed SiO_2**	
	**Incubation time (h)**	**2**	**6**	**2**	**6**
**Recovery (%)**	**Caco-2 monolayer**	101.55 ± 2.40	98.80 ± 1.49	98.78 ± 0.88	98.66 ± 1.48
	**FAE model**	99.22 ± 3.60	100.34 ± 1.20	98.99 ± 0.55	99.25 ± 2.12
**Transport (%)**	**Caco-2 monolayer**	0.03 ± 0.95	0.95 ± 0.01	0.04 ± 0.07	0.67 ± 0.01
	**FAE model**	1.07 ± 0.17	2.08 ± 0.01	0.54 ± 0.06	1.54 ± 0.01
	**Total**	1.10 ± 1.12	3.03 ± 0.02	0.58 ± 0.13	2.21 ± 0.02

Table S2. Composition of artificial lysosomal fluid (ALF).

pH	Composition (amounts/L)
4.5	sodium chloride (3.2 g), sodium hydroxide (6.0 g), calcium chloride dihydrate (128 mg), sodium phosphate (71 mg), sodium sulfate (39 mg), magnesium chloride hexahydrate (106 mg), formaldehyde (1 mL), citric acid (20.8 g), glycerin (59 mg), sodium citrate dihydrate (77 mg), sodium tartrate dihydrate (90 mg), 60% sodium lactate (65 μL), sodium pyruvate (86 mg), glycine (30.3 g)

Table S3. Compositions of simulated digestion fluids

Digestion fluids	pH	Compositions (amounts/L)
Saliva	6.8±0.1	potassium chloride (896 mg), potassium thiocyanate (200 mg), sodium phosphate monobasic dihydrate (1.1 g), sodium sulfate (570 mg), sodium chloride (298 mg), sodium bicarbonate (1.7 g), urea (200 mg), α-amylase (290 mg), uric acid (15 mg), mucin (25 mg)
Gastric fluid	1.3±0.1	sodium chloride (2.8 g), potassium chloride (824 mg), sodium dihydrogen phosphate dihydrate (332.8 mg), calcium chloride dihydrate (400 mg), urea (85 mg), D-(+)-glucose (650 mg), glucuronic acid (20 mg), glucosaminhydrochloride (330 mg), bovine serum albumin (1.0 g), pepsin (2.5 g), mucin (3.0 g)
Duodenal fluid	8.1±0.1	sodium chloride (7.0 g), sodium bicarbonate (3.4 g), potassium chloride (564 mg), magnesium chloride (22.7 mg), potassium dihydrogen phosphate (80 mg), 37% hydrochloride (180 μL), urea (100 mg), bovine serum albumin (1 g), pancreatin (9.0 g), lipase (1.5 g), calcium chloride dihydrate (200 mg)
Bile fluid	8.2±0.1	sodium chloride (5.3 g), sodium bicarbonate (5.8 g), potassium chloride (376 mg), 37% hydrochloride (150 μL), urea (250 mg), bovine serum albumin (1.8 g), bile (30.0 g), calcium chloride dihydrate (222 mg)
